# Do cortisol and psychological distress levels impact the effectiveness of immunotherapy in patients with metastasized melanoma? A pilot study

**DOI:** 10.1097/CMR.0000000000001035

**Published:** 2025-03-25

**Authors:** Chris Hinnen, Frederiek Tijssens, Emma von Haeseler, Sjoerd van de Berg, Ellen Kapiteijn

**Affiliations:** a Psycho-oncology, Leiden University Medical Centre, Leiden; b Department of Psychiatry and Medical Psychology, Spaarne Gasthuis, Haarlem; c Department of Clinical Chemistry, Erasmus Medical Centre, Rotterdam; d Medical Oncology, Leiden University Medical Centre, Leiden, the Netherlands

**Keywords:** cortisol, depression, immunotherapy, melanoma, stress

## Abstract

This pilot study investigates the relationship between endogenous cortisol and subjective distress and immunotherapy response in patients with advanced melanoma. Patients were asked to donate hair and complete questionnaires. This data was related to immunotherapy response, 3 and 6 months after start. Results from 21 patients were analyzed and showed that there was a significant relationship between depressive symptoms before start of immunotherapy and response 3 and 6 months after start of immunotherapy. Also, a higher baseline level of glucocorticoids was found to be significantly associated with a higher response rate 6 months after start of immunotherapy. The present pilot study warrants further investigation into the relationship between stress and the effectiveness of immunotherapy in patients with advanced melanoma.

## Introduction

Immunotherapy with checkpoint inhibitors in metastasized melanoma patients is nowadays a standard treatment option that is effective in approximately 40% of the cases [1]. Whether people respond to immunotherapy is mostly apparent after 3 and 6 months and, until date, often unpredictable although age, gender, lactate dehydrogenase (LDH), number of metastases and whether or not metastases are found in the brain, and WHO performance score are found to be associated with overall survival [2]. Identifying additional alterable factors differentiating responders from nonresponders after immunotherapy is important as it may direct the development of additional interventions and the allocation of scarce medical and financial resources.

Recently, scholars and researchers have suggested that chronic stress may impact the effectiveness of immunotherapy. For example, patient with advanced non-small cell lung cancer receiving immunotherapy who reported more stress were found to show a shorter overall and progression free survival [3]. The cause for this might be that chronic stress is known to impact the immune system by the release of glucocorticoids which suppresses immune reactions [4]. Consequently, chronic stress may weaken the efficacy of immunotherapy which is aimed at boosting the immune system. Indirect and preliminary evidence supporting the link between chronic stress and the effectiveness of immunotherapy is found in studies investigating the impact of exogenous steroids (e.g. glucocorticoids) on the effectiveness of immunotherapy in mouse [5,6] as well as in patients [7]. For example, in a study among patients with advanced melanoma it was found that a higher dose of steroids could negatively impact survival outcomes [8]. However, a recent meta-analyses concluded that the use of glucocorticoids for non-cancer related indications does not affect immunotherapy response and survival [9].

The aim of the present pilot study was to investigate whether chronic stress measured with both objective (hair-cortisol) and subjective (questionnaires) measures and immunotherapy response at 3 and 6 months after start of immunotherapy in patients with metastasized melanoma in order to determine whether this line of research is promising.

## Material and method

After informed consent and before start of immunotherapy, patients were asked to donate hair from the posterior vertex as close to the scalp as possible (100–150 hairs, 3 cm long) and complete questionnaires. Hair glucocorticoids was analyzed per centimeter, representing the first, second, and third month before start of immunotherapy. For a detailed description of how hair was analyzed see Mirzaian *et al*. [10]. The questionnaires consisted of the the 16-item (di)stress subscale of the *Four-Dimensional Symptom Questionnaire* and the 20-item Center for Epidemiological Studies Depression Scale (CES-D). Immunotherapy response was assessed during regular care, at 3 and 6 months after start of treatment with radiologic evaluations; either computed tomography (CT) or PET-CT scans or MRI brain.

The relationship between gender, clinical factors (LDH, WHO, number of locations with metastasis, whether or not there are metastases in the brain), hair glucocorticoids, and self-report questionnaires on the one hand and immunotherapy response [partial response (PR)/stable disease vs progressive disease (PD)] at 3 and 6 months on the other hand was analyzed using independent *t*-tests (with Levene’s test) for continuous variables and chi-square test for dichotomized variables. Pearson’s correlations between the main factors are also calculated. Given the small sample size only univariate analyses were performed. Missing variables were not imputed. The study was approved by Research Ethical Committee Leiden, The Hague and Delft, the Netherlands (P19.072) and no funding was available.

## Results

In total 29 patients received information about the study of whom 23 met inclusion criteria (i.e. diagnosed with metastasized melanoma, start of immunotherapy within a week) and signed informed consent. Thirteen (56.5%) were male and the mean age was 63.17 (9.76) years. After inclusion and completion of baseline measures, two patients showed hair-cortisol levels (>80 pg/mg) indicating the administration of exogenous glucocorticoids (e.g. hydrocortisone) and were excluded from further analyses. Correlations between the main variables are presented in Table 1. No correlations between hair cortisol and self-reported distress were found. Moreover, no differences were found between the stable and progressive group in WHO and LDH scores. On average patients had metastases in almost three locations with 7 patients having more than three metastases. The most common location with metastases were the lungs, 14 times. In 7 patients, metastases were localized in the brain. Patients with brain metastases had a 86% change of being in the progressive group.

**Table 1:** Correlations between the main variables

	LDH	Number of metastases	0–1 cm, pg/mg	1–2 cm, pg/mg	2–3 cm, pg/mg	0–3 cm, pg/mg	4DSQ	CES-D
Age	0.16	0.16	−0.04	−0.03	−0.11	−0.03	0.09	−0.22
LDH		0.20	−0.07	−0.02	−0.16	−0.05	0.33	−0.01
Number of metastases			−0.19	−0.29	−0.26	−0.22	0.42	0.33
0–1 cm, pg/mg				0.94**	0.90**	0.98**	−0.30	−0.27
1–2 cm, pg/mg					0.95**	0.99**	−0.21	−0.20
2–3 cm, pg/mg						0.97**	−0.21	−0.23
0–3 cm, pg/mg							−0.25	−0.24
4DSQ								0.65**

4DSQ, Four-Dimensional Symptom Questionnaire; CES-D, Center for Epidemiological Studies Depression Scale; LDH, lactate dehydrogenase.

** <0.001.

At 3 and 6 months after start of immunotherapy, respectively 12 (57%) and 13 (62%) patients showed PD with males showing a bigger change of being in the PD group at 6 months after start of immunotherapy (*P* < 0.01). Moreover, patients in the stable/PR group showed higher levels of hair cortisol before start of immunotherapy. Six months after start of treatment this difference was found to be significant. In addition, patients who showed PD at 3 or 6 months after start of immunotherapy reported higher scores on the CES-D questionnaire before start of immunotherapy (see Table 2).

**Table 2:** Differences between immunotherapy responders and nonresponders on baseline characteristics

		3 months after start			6 months after start		
Baseline characteristics	Total (21)	Stable disease/partial response (*N* = 9, 43%)	Progressive disease(*N* = 12, 57%)	*P* value	Stable disease/partial response (*N* = 8, 43%)	Progressive disease(*N* = 13, 62%)	*P* value
Gender							
Male	11	3	8	0.13	1	10	<0.01
Female	10	6	4		7	3	
WHO							
0	10	4	6	0.50	2	8	0.41
1	8	2	6		3	5	
Missing	3						
LDH, mean (SD)	214.74 (62.29)	219.56 (73.84)	210.4 (53.57)	0.76	231.38 (76.80)	202.64(49.73)	0.27
LDH < 250							
No	7	3	4	0.76	4	3	0.31
Yes	12	6	6		4	8	
Missing	2						
Number of metastases				0.19			0.42
Mean (SD)	2.86 (1.56)	2.33 (1.66)	3.25 (1.42)		2.50 (1.77)	3.08 (1.44)	
>3	7	2	5		2	5	
Metastasis brain				0.06			0.11
Yes	7	1	6		1	6	
No	14	8	6		7	7	
Hair cortisol							
0-1 cm, pg/mg, mean (SD)	3.23 (2.04)	4.07 (3.05)	2.74 (1.02)	0.18	4.67 (3.15)	2.57(.78)	0.03
1-2 cm, pg/mg, mean (SD)	2.81 (1.61)	3.33 (2.31)	2.44 (.85)	0.36	3.87 (2.26)	2.23(.76)	0.04
2-3 cm, pg/mg, mean (SD)	2.45 (1.56)	2.76 (2.26)	2.21 (.77)	0.56	3.50 (1.95)	1.82 (.88)	0.03
0-3 cm, pg/mg, mean (SD)	2.80 (1.65)	3.39 (2.49)	2.25 (.84)	0.25	4.01 (2.42)	2.24 (.74)	0.03
4DSQ, total (SD)	6.10 (5.19)	4.33 (3.39)	7.42 (6.01)	0.18	5.713 (2.90)	6.69(6.24)	0.52
CES-D, total (SD)	8.65 (6.01)	4.63 (3.62)	11.33 (5.87)	0.01	3.86 (3.13)	11.23 (5.63)	< 0.01

4DSQ, Four-Dimensional Symptom Questionnaire; CES-D, Center for Epidemiological Studies Depression Scale; LDH, lactate dehydrogenase.

## Discussion

In the present pilot study, we did find a statistically significant association between endogenous glucocorticoids and immunotherapy response at 6 months after start of immunotherapy. However, in contrast to our expectations, a somewhat higher baseline level of glucocorticoids was found to have a beneficial effect on response. This may support the notion of a more complex, dose dependent, and possible positive association between cortisol and immune system activation [11]. Another mechanism linking stress with immune functioning, that found recent support, stresses the exacerbating impact of catecholamines like adrenaline and noradrenaline on T-cell exhaustion [12]. Adding beta-blockers to immunotherapy may have a beneficial impact on the effectiveness of immunotherapy in patients with advanced melanoma [13].

It should be noted that the average hair cortisol levels found in the present study were relatively low and largely fall within the reference range [14]. Moreover, patients who were in the progressive group at 3 or 6 months reported more depressive symptoms before start of immunotherapy than patients who showed stable disease at these time points. This finding is in accordance with a previous study among patients with high risk stage III melanoma [15] showing that emotional distress was associated with clinical outcomes. The mechanism underlying this association remain largely unclear as both adrenergic-driven or glucocorticoid-driven mechanisms have not found much support [16]. In the present study we also found no support for cortisol mediating this relationship as no association was found between emotional distress and hair cortisol levels. Not finding an association between hair cortisol and subjective stress measures is however not uncommon [17].

Given the small sample size the findings should be treated with caution but warrant additional research. When replicated, possible mechanisms should be investigated. Moreover, due to the sample size we were not able to investigate covariates and conduct multivariate analyses. Additional information such as subtype of melanoma were not included.

The present pilot study is the first to investigate the relationship between endogenous cortisol and subjective distress and immunotherapy response in patients with advanced melanoma. Although the small sample size limits the interpretation of the results, it warrants further investigation. If our results are confirmed, investigating the mechanisms relating psychological distress with immunotherapy outcomes and the effectiveness of preemptive measures to reduce (emotional) stress before and during immunotherapy is needed.

## Acknowledgements

C.H. and E.K. contributed in the design and writing/drafting. F.T. and E.H. contributed in the data collection. C.H. and S.B. contributed in the analyses.

Ethical approval was obtained from Ethical Committee Leiden, The Haque, The Netherlands (P19.072).

Data availability: on request.

### Conflicts of interest

There are no conflicts of interest.
